# Loco-Regional Anesthesia for Pain Management in Robotic Thoracic Surgery

**DOI:** 10.3390/jcm13113141

**Published:** 2024-05-27

**Authors:** Luigi La Via, Marco Cavaleri, Alberto Terminella, Massimiliano Sorbello, Giacomo Cusumano

**Affiliations:** 1 Department of Anesthesia and Intensive Care, Azienda Ospedaliera Universitaria Policlinico “G. Rodolico-San Marco”, 95123 Catania, Italy; cavalerimarco@hotmail.it; 2 Department of Thoracic Surgery, Azienda Ospedaliera Universitaria Policlinico “G. Rodolico-San Marco”, 95123 Catania, Italy; a.terminella@ao-ve.it (A.T.); giacomare55@hotmail.com (G.C.); 3 Faculty of Medicine and Surgery, University “Kore”, 94100 Enna, Italy; maxsorbello@gmail.com; 4 Department of General Surgery and Medical-Surgical Specialties, University of Catania, 95123 Catania, Italy

**Keywords:** thoracic surgery, block, epidural, intercostal, paravertebral, myasthenia gravis, erector spinae plane, local anesthesia

## Abstract

Robotic thoracic surgery is a prominent minimally invasive approach for the treatment of various thoracic diseases. While this technique offers numerous benefits including reduced blood loss, shorter hospital stays, and less postoperative pain, effective pain management remains crucial to enhance recovery and minimize complications. This review focuses on the application of various loco-regional anesthesia techniques in robotic thoracic surgery, particularly emphasizing their role in pain management. Techniques such as local infiltration anesthesia (LIA), thoracic epidural anesthesia (TEA), paravertebral block (PVB), intercostal nerve block (INB), and erector spinae plane block (ESPB) are explored in detail regarding their methodologies, benefits, and potential limitations. The review also discusses the imperative of integrating these anesthesia methods with robotic surgery to optimize patient outcomes. The findings suggest that while each technique has unique advantages, the choice of anesthesia should be tailored to the patient’s clinical status, the complexity of the surgery, and the specific requirements of robotic thoracic procedures. The review concludes that a multimodal analgesia strategy, potentially incorporating several of these techniques, may offer the most effective approach for managing perioperative pain in robotic thoracic surgery. Future directions include refining these techniques through technological advancements like ultrasound guidance and exploring the long-term impacts of loco-regional anesthesia on patient recovery and surgical outcomes in the context of robotic thoracic surgery.

## 1. Introduction

Robotic surgery has revolutionized the field of minimally invasive surgery, allowing for enhanced precision, improved range of motion, and better visualization of the surgical field [1,2] while offering equivalent surgical radicality than open surgery [3]. In particular, thoracic surgery particularly benefits from these advancements [4,5]. This approach typically results in less postoperative pain, reduced blood loss, shorter hospital stays, and quicker recovery compared to the traditional open surgery [6]. Additionally, the cosmetic benefits of robotic surgery are highly preferred by patients. Furthermore, video-assisted thoracoscopic or robot-assisted techniques are commonly employed in cases requiring pleurectomies for pleural dissemination of disease [7]. Despite these benefits, effective pain management remains a critical component of postoperative care. Pain after thoracic surgery can be severe and, if inadequately controlled, may lead to adverse effects such as impaired respiratory function, increased risk of pulmonary complications, delayed mobilization, and prolonged recovery [8]. Loco-regional anesthesia techniques have gained popularity in managing perioperative pain for thoracic surgeries, as they can provide superior pain control, reduce systemic opioid consumption, and potentially improve postoperative outcomes [9]. The use of loco-regional anesthesia techniques, such as local infiltration (LIA), thoracic epidural anesthesia (TEA), paravertebral block (PVB), intercostal nerve block (INB) and erector spinae plane block (ESPB), can offer distinct advantages during robotic thoracic procedures. However, the choice of technique must consider the patient’s clinical status, the anticipated complexity of the surgery, and the potential risks and benefits of each method. This comprehensive review will focus on the current evidence, techniques, and implications of these loco-regional anesthesia methods in the context of robotic thoracic surgery, aiming to provide a narrative overview for anesthesiologists and thoracic surgeons alike. The goal is to outline the role of each technique, discuss their respective advantages and challenges, and explore how they can be effectively integrated into clinical practice to improve patient care in robotic thoracic surgery.

## 2. Materials and Methods

A comprehensive literature search of online databases including PubMed, Scopus, MEDLINE, EMBASE, Cochrane Library, Google Scholar and Web of Science was conducted to identify studies relevant to loco-regional anesthesia techniques used in robotic thoracic surgery. We included three groups of terms in our searches. The first one included “anesthesia OR block OR blocks OR regional OR spinal OR epidural OR local OR loco-regional OR anesth*”. The second one was composed by “Thymectomy OR thymus OR lung OR mediastinum OR thoracic OR thym* OR thorac*”. Finally, the last one included “robot OR robotic OR Da Vinci”. The search strings were adapted for each database to match its specific indexing system and search algorithms. The search was designed to include all relevant articles published in English up to March 2024, with no restrictions on the publication date to encompass the evolution of both surgical and anesthetic techniques over time.

### Study Selection

The inclusion criteria were prospective and retrospective studies, randomized controlled trials, observational studies, case series, and review articles that discussed the use and outcomes of loco-regional anesthesia techniques in the context of robotic thoracic surgery. Exclusion criteria encompassed articles not focused on the designated surgical or anesthetic techniques, case reports with limited generalizability, and studies with incomplete data. Two independent reviewers screened titles and abstracts for relevance. The full texts of potentially relevant articles were then retrieved and examined in detail. Disagreements between reviewers were resolved through discussion and, if necessary, consultation with a third reviewer.

## 3. Local Infiltration

Local infiltration (LIA) involves the direct injection of a local anesthetic solution into the tissue surrounding the surgical site [10]. It’s simple and can be performed by the surgeon, but may not provide complete analgesia for deeper structures. Studies show its effectiveness in reducing immediate postoperative pain [11].

### 3.1. Technique

The application of LIA in robotic thoracic surgery involves administering the anesthetic agent either preoperatively or intraoperatively by the surgeon at key anatomical landmarks associated with the planned incision sites and potential pain generators [10,12].

### 3.2. Advantages in Robotic Thoracic Surgery

The advantages of LIA are numerous. It is simple and quick to perform and does not require advanced equipment or extensive training [10]. Additionally, LIA carries a low risk of systemic toxicity and can be administered with minimal disruption to the surgical workflow. The technique also avoids the potential for the hemodynamic changes often seen with neuraxial or central blocks [12]. Several studies have demonstrated the efficacy of LIA in reducing immediate postoperative pain and opioid consumption. For instance, a randomized controlled trial by Sihoe et al. found that pre-emptive wound infiltration with a local anesthetic may reduce post-operative wound pain in thoracic procedures [13]. Moreover, current evidence supports the use of LIA as an adjunct to multimodal analgesia for thoracic surgery procedures performed in high-risk patients [14,15,16]. In this context, local anesthetic can be infiltrated around the port sites and along the intercostal spaces that may be affected during the procedure [17]. This can provide satisfactory postoperative analgesia, facilitating early patient mobilization and reducing the length of hospital stay [18]. However, surgical procedures performed exclusively using LIA should be minimally invasive in order to allow the patients to keep spontaneous breathing under a mild sedation.

### 3.3. Limitations and Challenges

LIA is not without limitations. Its duration of action is finite, and it may not provide complete analgesia for deeper surgical pain or visceral pain that can occur following robotic thoracic surgery. Additionally, there is a risk of local anesthetic systemic toxicity (LAST) if large volumes of local anesthetic are used, especially in the highly vascular thoracic area [19]. Other possible complications include allergic reactions, needle injury, bleeding and hematoma.

## 4. Thoracic Epidural Anesthesia

Thoracic epidural anesthesia (TEA), also known as peridural anesthesia, involves the injection or continuous infusion of local anesthetics, often combined with analgesics, into the epidural space surrounding the dura mater of the spinal cord [20]. This method is frequently used for pain management in thoracic surgical procedures due to its effective regional analgesia [21].

### 4.1. Technique

The procedure is typically performed with the patient in a sitting or lateral decubitus position to open the intervertebral spaces. After sterile preparation, a needle is introduced into the epidural space using loss-of-resistance technique, either with saline or air. Once the epidural space has been identified, a catheter is threaded through the needle, which is then removed, leaving the catheter in place for continuous medication administration.

### 4.2. Advantages in Robotic Thoracic Surgery

The major advantage of TEA is the ability to provide segmental analgesia, which can be tailored to the surgical site [20]. During thoracic surgery, TEA can provide effective analgesia without further intravenous opioids requirements [21]. This targeted pain control can facilitate early extubation, early mobilization and reduce pulmonary complications [22]. Also, the continuous infusion through an epidural catheter can maintain pain control for an extended period postoperatively. Kawagoe et al. [23] aimed at comparing the postoperative pain scores and opioid requirements in patients anesthetized with either general anesthesia alone or combined general anesthesia and TEA. In this study, authors found that TEA provided better analgesia after robotic thymectomy in terms of less pain scores, less rescue analgesic requirements, and similar side effect profiles. In fact, TEA can be particularly effective for managing the pain associated with port site incisions and the dissection of the thymus gland. Moreover, Kusano et al. [24] performed a retrospective study comparing the effect of general anesthesia alone, general anesthesia combined with TEA, or general anesthesia combined with ultrasound-guided thoracic blocks. Their results showed that TEA provided better analgesia after robot-assisted thoracic surgery for mediastinal disease than general anesthesia alone, as indicated by lower pain scores and fewer rescue analgesic requirements. For these reasons, TEA combined with general anesthesia is commonly considered as the preferred anesthetic technique for thoracic surgery [20]. However, a prospective study by Fiorelli et al. [25] showed that the mini-invasive approach granted by thoracoscopic and robotic thoracic surgery is associated with lower pain in the postoperative period, thus possibly suggesting less invasive approaches for analgesia. For this reason, current PROSPECT guidelines no longer recommend TEA for thoracoscopic surgery [26]. In conclusion, since clear evidence-based recommendations for optimal postoperative analgesia are still lacking in robotic thoracic surgery, there can be no universal recommendation that fits all centers and patients. In this context, TEA might be the most effective analgesia procedure for perioperative pain control in robotic-assisted surgery for patients with pulmonary risk factors [22,27].

### 4.3. Limitations and Challenges

There are potential complications and considerations specifically associated with the use of TEA in robotic thoracic surgery [28]:

- Hemodynamic Instability: The administration of local anesthetics into the epidural space can lead to sympathetic nerve blockade, which may result in vasodilation and hypotension. Careful monitoring and management of the patient’s fluid status and hemodynamics are required to prevent and treat hypotension [29,30].
- Respiratory Compromise: TEA can affect intercostal muscles and, to a lesser extent, the diaphragm, which may reduce the patient’s ability to breathe deeply and cough effectively, potentially leading to atelectasis or pneumonia, especially in patients with pre-existing pulmonary conditions.
- Infection: The introduction of a catheter for epidural anesthesia can increase the risk of infection, including epidural abscess or discitis.
- Epidural Hematoma: This is a rare but serious complication, particularly in patients with coagulopathy or those on anticoagulant therapy. It can lead to spinal cord compression and requires immediate recognition and surgical intervention.
- Catheter-Related Issues: Catheter migration, kinking, or dislodgement can result in inadequate analgesia. Proper securing of the catheter and monitoring of its function are important.
- Technical Difficulties: The correct placement of an epidural catheter can be technically challenging, and inadvertent dural puncture may occur, potentially leading to a post-dural puncture headache.
- Need for close monitoring: Patients receiving peridural anesthesia require close monitoring since the epidural catheter is in place. This includes regular assessments of pain control, motor and sensory block levels, hemodynamic parameters, and potential side effects or complications from the infusion. The need for close monitoring may also delay discharge after robotic thoracic surgery.

It’s important to note that while these complications are potential risks, the incidence is generally low, and TEA is considered safe and effective when performed by experienced clinicians [31].

## 5. Paravertebral Block

The paravertebral block (PVB) is a regional anesthetic technique that targets the nerves supplying the chest wall and intra-thoracic structures. It has gained popularity in thoracic anesthesia, particularly for minimally invasive procedures like robotic thymectomy [32]. By depositing local anesthetic adjacent to where the spinal nerves emerge from the intervertebral foramina, PVB achieves ipsilateral somatic and sympathetic nerve blockade.

### 5.1. Technique

The technique for a thoracic PVB can be performed using either a landmark-based approach or with the aid of ultrasound guidance, which has increased in popularity due to its higher accuracy and safety profile [8,32,33]. The patient is usually in a sitting or lateral decubitus position. After sterile preparation, a needle is introduced perpendicular to the back, just lateral to the spinous process at the desired thoracic level, typically at the level of T2–T4 for thoracic robotic procedures [34]. The endpoint for the needle is the paravertebral space, which can be identified by a loss of resistance or using ultrasound to visualize the needle’s approach to the parietal pleura. The thoracic paravertebral space is a wedge-shaped area located on either side of the vertebral column. It contains the spinal nerves, rami communicantes, and sympathetic chain. When local anesthetic is injected into this space, it can diffuse around the thoracic spinal nerves, leading to unilateral segmental anesthesia of the chest wall and some visceral structures [34]. The extent of the block can be tailored by the choice of injection level and volume of anesthetic used.

### 5.2. Advantages in Robotic Thoracic Surgery

Very few studies have been performed on the efficacy of PVB in robotic thoracic surgery. However, the effects of PVB have been investigated following video-assisted thoracoscopic surgery [35] and open thoracic surgery, where it appears to offer an equivalent level of analgesic effect, with a more favourable side-effect profile, as compared to TEA [36,37] or other blocks of the thoracic wall [38]. In fact, PVB is associated with reduced postoperative pain as well as morphine and antalgic drugs administration [39]. Also, PVB has shown to improve early mobilization after robotic thoracic surgery, thus requiring a shorter length of hospital stay [39]. Moreover, PVB does not affect hemodynamic stability, showing a safer profile as compared to TEA [40], especially in cardiopathic patients. Lastly, PVB preserves pulmonary function by avoiding the bilateral motor blockade that is often associated with TEA, which is essential in patients with pre-existing respiratory conditions [40]. For these reasons, according to the PROSPECT guidelines, PVB can be used as first line block for video-assisted thoracoscopic surgery [35].

### 5.3. Limitations and Challenges

Despite its efficacy, there are limitations when using PVB for robotic thoracic surgery. One significant limitation is the technical difficulty and variability in anatomical landmarks, which can lead to inconsistent block success rates [34]. PVB relies on the precise placement of local anesthetic near the thoracic paravertebral space, adjacent to where the spinal nerves emerge from the intervertebral foramina, and inaccurate needle placement can result in inadequate analgesia or failed blocks. Furthermore, there is a risk of complications such as pneumothorax, vascular puncture, or nerve injury, which can be particularly concerning given the close proximity to major vessels and the pleura [41]. The advent of ultrasound-guided PVB has mitigated some risks by improving visualization, but it requires specific expertise and is not universally available [42]. Additionally, the hemodynamic alterations from sympathectomy associated with PVB, such as hypotension and bradycardia, may pose a risk in certain patient populations, and thus careful patient selection is necessary [43]. In robotic thoracic surgery, where patient positioning and the surgical setup can be complex, accessibility for PVB may be limited once the patient is positioned, potentially necessitating the block to be performed prior to confirming the final surgical position [44]. Moreover, the extended duration of robotic procedures may exceed the analgesic window provided by single-injection PVB, necessitating continuous catheter techniques, which add complexity and maintenance requirements [45].

## 6. Intercostal Nerve Block

Intercostal nerve block (INB) is a regional anesthesia technique used for pain management in various thoracic and abdominal procedures. The intercostal nerves arise from the anterior rami of the thoracic spinal nerves and run along the subcostal groove beneath each rib [46]. Blocking these nerves can provide effective analgesia for procedures involving the chest wall and upper abdomen.

### 6.1. Technique

The INB technique involves the injection of local anesthetic near the intercostal nerve along its pathway. This has been traditionally done by identifying the relevant intercostal space and using anatomical landmarks to deposit the anesthetic just under the rib, close to the intercostal nerve. However, the use of ultrasound guidance for INB has several advantages over traditional landmark-based techniques [47,48,49]. In fact, ultrasound allows for real-time visualization of the needle, the intercostal space, and the surrounding structures, including the pleura, muscles, and vessels. Also, ultrasound guidance is associated with increased safety and improved success rates, as well as the reduced volume of local anesthetic used. The INB can also be performed by the surgeon, who can inject the local anesthetic through direct visualization of the intercostal space during surgery.

### 6.2. Advantages in Robotic Thoracic Surgery

The primary advantage of INBs is the provision of targeted analgesia with a relatively low volume of local anesthetic. This can lead to improved pain control, reduced systemic opioid requirements, and potentially a quicker recovery and discharge from the hospital as compared to general anesthesia alone. In a recent meta-analysis by Guerra-Londono et al. [50], the use of INB was associated with lower static pain and lower dynamic pain during the first 24 h as compared to systemic analgesia alone for thoracic surgery. Also, single-injection INB was clinically noninferior to TEA in terms of postoperative analgesia. However, INB was significantly inferior to PVB in terms of dynamic pain, and it requires more opioid consumption in the postoperative period as compared to TEA and PVB [50]. Moreover, a recent randomized controlled trial [38] studied the analgesic effect of ultrasound-guided INB and single-injection erector spinae plane block (ESPB) in comparison with multiple-injection PVB after thoracoscopic surgery. This study showed that INB and single-injection ESPB were equally effective in reducing pain after thoracoscopic surgery. However, ultrasound-guided multiple-injection PVB provided superior analgesia to INB and single-injection ESPB [38]. This evidence suggests that INB may be most beneficial for cases in which TEA and PVB are not indicated.

### 6.3. Limitations and Challenges

INB has several limitations when applied to robotic thoracic surgery. Firstly, the duration of analgesia is often limited to a few hours, which may be insufficient for the postoperative pain that extends beyond the immediate perioperative period, necessitating repeated blocks or alternative analgesic strategies [46]. Secondly, the potential for systemic local anesthetic toxicity is increased with INB due to the high vascularity of the intercostal spaces, raising concerns when multiple levels are blocked or when large volumes of local anesthetic are used [51]. Additionally, the risk of pneumothorax, while relatively low, is a serious consideration, particularly when the block is performed without ultrasound guidance [52]. Performing INB in patients under general anesthesia also eliminates the feedback mechanism of pain upon pleural puncture, potentially increasing the risk of pneumothorax without immediate recognition [53]. Moreover, the patient positioning required for robotic thoracic surgeries can make access to intercostal spaces challenging, complicating the administration of INB after the patient has been positioned for surgery [54]. Furthermore, the somatic-only analgesia provided by INB may be insufficient for the visceral pain component that can be present following thoracic procedures [55].

## 7. Erector Spinae Plane Block

The Erector Spinae Plane Block (ESPB) is a relatively new regional anesthesia technique that has gained popularity for providing both analgesia for the chest wall and the visceral thoracic pain. It targets the dorsal rami of spinal nerves, offering a wide sensory block. This block is particularly advantageous for thoracic procedures due to its simplicity and safety profile. According to the PROSPECT guidelines, ESPB is the first line block along with PVB for video-assisted thoracoscopic surgery [35].

### 7.1. Technique

The technique involves the injection of local anesthetic in the fascial plane deep to the erector spinae muscle and superficial to the transverse processes of the vertebrae. Typically, the block is performed under ultrasound guidance which allows for real-time visualization of the anatomical structures and the local anesthetic spread. The ultrasound transducer is placed in a longitudinal orientation over the thoracic spine, identifying the relevant transverse process, and a needle is inserted in a cranial-to-caudal direction until the tip lies deep to the erector spinae muscle and superficial to the transverse process. A local anesthetic, is then injected, and it spreads craniocaudally and ventrally, to block the dorsal and ventral rami of the spinal nerves [56].

### 7.2. Advantages in Robotic Thoracic Surgery

ESPB has been shown to provide effective analgesia for thoracic surgical procedures, including thoracotomies and video-assisted thoracoscopic surgeries [38,57,58,59]. Its advantages include the simplicity of the technique, a potentially lower risk of complications such as pneumothorax, and the provision of both somatic and visceral analgesia [60]. However, as with any regional technique, there is a learning curve, and knowledge of thoracic paravertebral anatomy is essential for safe and effective block performance [61]. To date, evidence supporting the use of ESPB in robotic surgery is very limited and of low-moderate quality. A recent retrospective study by Durey et al. [62] showed that ESPB is associated with less post-operative static and dynamic pain at 24 h than PVB after robot-assisted thoracic surgery for lung cancer, with a high safety profile. Moreover, a randomized controlled trial [63] on sixty adult patients undergoing minimally invasive thoracic surgery compared a single-shot ESPB to serratus anterior plane block before surgery on the quality of patient recovery at 24 h. In this study, ESPB provided superior quality of recovery, lower morbidity, and better analgesia after robotic surgery. Given the relatively low duration of analgesia as compared to the long-lasting pain following robotic thoracic surgery, a continuous ESPB using a catheter infusion of local anesthetic might be an effective solution [64]. However, very few evidence exist on the efficacy and safety of this approach, thus requiring further studies.

### 7.3. Limitations and Challenges

Given that robotic thoracic surgery often requires patient positioning that may limit access to the back where the ESP block is placed, the block typically needs to be performed before the patient is positioned for surgery, which may affect the planning of the operating room workflow [65]. Additionally, the ESP block has been reported to provide variable levels of visceral analgesia, which could be a limitation for surgeries with significant visceral pain [66]. The block’s duration may also be insufficient for the extended postoperative pain period associated with complex robotic procedures, potentially necessitating continuous catheter techniques or supplemental analgesia [57]. Furthermore, while the ESP block is generally considered safe, there is a risk of local anesthetic systemic toxicity (LAST) due to the potential for vascular uptake of the local anesthetic, particularly when higher volumes are used [60]. The block’s efficacy can also vary depending on the level at which it is performed and the volume of local anesthetic used, which requires precise technique and knowledge of sonoanatomy for optimal results [67]. These limitations underscore the importance of a comprehensive, multimodal analgesic approach that may include the ESPB as one component in the management of postoperative pain in patients undergoing robotic thoracic surgery.

## 8. Serratus Anterior Plane Block

The serratus anterior plane block (SAPB) is a regional anesthetic technique that provides analgesia for thoracic surgical procedures by targeting the lateral cutaneous branches of the intercostal nerves.

### 8.1. Technique

The technique involves depositing local anesthetic in the plane either above (superficial SAPB) or below (deep SAPB) the serratus anterior muscle [68]. Superficial SAPB is typically performed by placing the ultrasound transducer in the midaxillary line at the level of the fourth or fifth rib and injecting local anesthetic above the serratus anterior muscle. Deep SAPB requires the local anesthetic to be placed beneath the serratus anterior muscle, which can provide more profound chest wall analgesia. The block can be performed preoperatively or intraoperatively, with ultrasound guidance ensuring precise anesthetic placement and potentially reducing the risk of complications. SAPB has been shown to provide effective analgesia for thoracotomies, rib fractures, and breast surgery, with the advantage of sparing the patient from the potential risks associated with more invasive techniques like thoracic epidurals [69]. However, the block may not fully cover the anterior and medial aspects of the thorax, and its duration may be limited, requiring repeated administration or catheter placement for continuous infusion [70].

### 8.2. Advantages in Robotic Thoracic Surgery

SAPB presents several advantages when utilized for robotic thoracic surgery. Firstly, it is a relatively straightforward and safe regional anesthesia technique that can be performed under ultrasound guidance, minimizing the risk of pneumothorax and vascular injury often associated with more invasive procedures like thoracic epidural or paravertebral blocks [69]. The SAPB can offer effective analgesia for lateral thoracic wall incisions, including those for robotic port access, by anesthetizing the lateral cutaneous branches of the intercostal nerves from T2 to T9 [70]. This can result in reduced intraoperative opioid consumption and improved postoperative pain control, which may facilitate earlier ambulation, reduced hospital stay, and faster recovery [68]. These advantages seem comparable to the ones reported with the use of INB [71]. However, the combination of SAPB and PVB does not seem to provide better analgesia as compared to PVB alone [72]. Another advantage provided by SAPB is that it can be performed with the patient in a supine position, which aligns with the positioning required for robotic thoracic procedures, thereby streamlining the workflow [73]. The block has also been associated with a low incidence of hemodynamic instability, making it a suitable option for patients with contraindications to neuraxial blocks [74]. Furthermore, the SAPB can be used as part of a multimodal analgesia strategy, complementing systemic analgesics and reducing the need for postoperative opioids, thereby limiting opioid-related side effects [75]. Collectively, these advantages make SAPB a valuable adjunct in the anesthetic management of patients undergoing robotic thoracic surgery.

### 8.3. Limitations and Challenges

SAPB, while useful for managing postoperative pain in thoracic surgery, encounters certain limitations when applied to robotic thoracic procedures. One key limitation is the potential inadequacy to provide complete analgesia for the complex innervation of the thorax involved in robotic surgeries, which often necessitates a multimodal analgesic approach [76]. The block may not fully cover the anterior and medial cutaneous branches of the intercostal nerves, which can result in insufficient analgesia for incisions or port sites used in robotic surgeries [73]. Additionally, the dynamic movement of the chest wall during robot-assisted procedures may impact the efficacy of the SAPB due to displacement of the local anesthetic or altered spread within the serratus anterior plane [74]. The duration of analgesia provided by a single-shot SAPB might not be sufficient for the postoperative period following lengthy robotic surgeries, potentially requiring the placement of a catheter for continuous infusion, which adds complexity and risk of catheter-related complications [76]. Moreover, the technical proficiency required to perform SAPB under ultrasound guidance and the need for patient repositioning during robotic surgery can pose logistical challenges in the operating room [70]. Lastly, while rare, there are risks associated with SAPB, such as local anesthetic systemic toxicity (LAST) and injury to surrounding structures, which must be considered when opting for this analgesic technique [69].

## 9. Local Anesthetics

For local wound infiltration, commonly used agents include lidocaine, bupivacaine, or ropivacaine, owing to their efficacy and duration of action [77]. These agents may be used alone or in combination with adjuvants such as epinephrine, which prolongs the duration of analgesia by reducing local blood flow and systemic absorption of the anesthetic [11]. Similarly, for anesthetic blocks such as TEA, PVB, INB, SAPB and ESPB, a variety of local anesthetics has been studied, including lidocaine, bupivacaine, and ropivacaine. The choice of anesthetic depends on the desired duration of analgesia, with longer-acting agents like bupivacaine and ropivacaine preferred for postoperative pain management due to their prolonged effect. All these anesthetics may be combined with adjuvants, such as dexamethasone, alpha-2 agonists or opioids, in order to prolong their effect. Moreover, recent studies also evaluated the effect of liposomal bupivacaine for INB [78,79]. In fact, liposomal bupivacaine offers the potential to provide prolonged blockade of intercostal nerves (72 to 96 h), as compared to bupivacaine alone [80], or in combination with epinephrine [78].

## 10. Discussion

Table 1 provides an overview of all the possible loco-regional anesthetic techniques discussed, including advantages and disadvantages.

The future of loco-regional anesthesia techniques in robotic thoracic surgery holds significant promise for enhancing patient outcomes through precision medicine and technological integration. As the demand for minimally invasive surgical approaches grows, so too does the need for equally minimally invasive anesthetic techniques that provide effective analgesia, minimize systemic opioid use, and facilitate rapid postoperative recovery [81].

The advancement of ultrasound technology is expected to further refine nerve localization for blocks, potentially in combination with augmented reality interfaces for real-time, three-dimensional guidance [82]. Moreover, new techniques can be studied in order to provide a more tailored analgesia according to the surgery performed. One example could be provided by the retrolaminar block (RLB), which is another ultrasound-guided technique that has been reported to provide thoraco-abdominal wall analgesia similarly to ESPB but with no spread of local anesthetic into the intercostal and paravertebral spaces [57,83]. RLB has been associated with optimal pain control for rib fracture and in several laparoscopic retroperitoneal procedures [84,85]. However, to date little evidence exist on its role during thoracoscopic surgery [86].

The development of long-acting local anesthetics and the use of adjuvants may extend the duration of single-shot nerve blocks [87], as reported in other settings [88]. Additionally, the exploration of continuous infusion techniques through catheter placement could provide sustained pain control postoperatively, aligning with the extended recovery period associated with complex robotic procedures [89]. There is also a growing interest in the investigation of the impact of loco-regional anesthesia on cancer recurrence and the immune system, which may prove particularly relevant in thoracic oncology surgery [90]. The integration of loco-regional anesthesia with enhanced recovery after surgery (ERAS) protocols is likely to be a focus, aiming to optimize perioperative care pathways and improve surgical outcomes [91]. Lastly, emerging formulations of local anesthetics, such as liposomal bupivacaine, which provide extended release of the drug, may overcome some of the limitations of traditional LIA by providing longer-lasting analgesia. Research into the optimal drug combinations, concentrations, and volumes for LIA specifically in robotic thymectomy will continue to refine the application of this technique. As research continues to unfold, personalized analgesia, where loco-regional techniques are tailored to the individual’s genetic profile, surgical procedure, and pain response, could become a reality, marking a significant evolution in the field of anesthesiology and patient-centered care. The present review is limited by its narrative design and the lack of large randomized studies comparing the various loco-regional anesthesia techniques in the specific setting of the robotic thoracic surgery.

## 11. Future Directions

The future of loco-regional anesthesia in robotic thoracic surgery is promising, with advancements in technology and precision medicine expected to enhance patient outcomes. As minimally invasive surgical approaches continue to gain popularity, there is a growing need for equally minimally invasive anesthetic techniques that provide effective analgesia, reduce systemic opioid use, and promote rapid postoperative recovery. The development of ultrasound technology is anticipated to further refine nerve localization for blocks such as the serratus anterior plane and erector spinae plane blocks, potentially combined with augmented reality interfaces for real-time, three-dimensional guidance. Long-acting local anesthetics and the use of adjuvants may extend the duration of single-shot nerve blocks, while continuous infusion techniques through catheter placement could provide sustained pain control postoperatively, aligning with the extended recovery period associated with complex robotic procedures. Furthermore, there is growing interest in investigating the impact of loco-regional anesthesia on cancer recurrence and the immune system, which may prove particularly relevant in thoracic oncology surgery. The integration of loco-regional anesthesia with enhanced recovery after surgery (ERAS) protocols is likely to be a focus, aiming to optimize perioperative care pathways and improve surgical outcomes. Emerging formulations of local anesthetics, such as liposomal bupivacaine, which provide extended release of the drug, may overcome some of the limitations of traditional local infiltration anesthesia by providing longer-lasting analgesia. Research into the optimal drug combinations, concentrations, and volumes for local infiltration anesthesia specifically in robotic thoracic surgery will continue to refine the application of this technique. As research continues to unfold, personalized analgesia, where loco-regional techniques are tailored to the individual’s genetic profile, surgical procedure, and pain response, could become a reality, marking a significant evolution in the field of anesthesiology and patient-centered care.

## Figures and Tables

**Table 1:** Overview of advantages and limitations of the loco-regional anesthesia techniques discussed.

Loco-Regional Technique	Advantages	Limitations
Local Wound Infiltration	Easy to perform No advanced equipment or extensive training required Low risk of LAST	Limited duration of analgesic effect No deeper surgical field or visceral pain coverage Risk of local bleeding
Thoracic Epidural Analgesia	Provide effective segmental analgesia and can be tailored to the surgical field Continuous infusion over the surgery.	Hemodynamic instability Possible respiratory compromise, infections, neuraxial hematoma.
Paravertebral Block	Effective analgesic option (similar to thoracic epidural) Low risk of hemodynamic and respiratory compromise US guidance	Technically difficult to perform Limited duration of analgesic effect Risk of vessel and pleura lesions
Intercostal Nerve Block	Easy to perform Limited LA volume US guidance.	No visceral analgesia Moderate risk of LAST for multiple injection
Erector Spinae Plane Block	Simple and safe Extended segmental analgesia provided with proper LA volume US guidance Continuous infusion	Limited duration of analgesic effect Variable level of visceral analgesia Low-moderate risk of LAST
Serratus Anterior Plane Block	Simple and safe Extended duration of analgesia with single bolus Continuous infusion US guidance.	Potential limited coverage of surgical field in thoracic robotic surgery No visceral analgesic effects Low-moderate risk of LAST

LAST: Local anesthetic systemic toxicity; US: Ultrasound; LA: local anesthetic.

## Data Availability

No new data were created or analyzed in this study. Data sharing is not applicable to this article.
